# Eosinophilic Esophagitis: The Role of Steroids and the Dose, Duration, and Delivery of Steroid Therapy

**DOI:** 10.7759/cureus.58343

**Published:** 2024-04-15

**Authors:** Sruthi Priyavadhana Ramanan, Bipneet Singh, Sri Harshini Gandhamaneni, Ibrahim Sange

**Affiliations:** 1 Research, California Institute of Behavioral Neurosciences & Psychology, Fairfield, USA; 2 Medicine/Surgery, Saveetha Medical College, Chennai, IND; 3 Internal Medicine, Henry Ford Health System, Jackson, USA; 4 Internal Medicine, Saveetha Medical College, Chennai, IND; 5 Internal Medicine, Karamshibhai Jethabhai Somaiya Medical College, Hospital & Research Center, Mumbai, IND

**Keywords:** eosinophilic esophagitis, steroids, budesonide, ciclesonide, fluticasone

## Abstract

Eosinophilic esophagitis (EoE) is a chronic immune-mediated condition characterized by the eosinophil infiltration of the esophagus (>15 per high power field). Recently, there has been an increase in both the incidence and prevalence of the disease. The common modalities of treatment are dietary modification, proton pump inhibitors, and steroids. However, the United States Food and Drug Administration has not approved any drugs for the treatment of EoE. This review has discussed the role of steroids in the treatment of EoE, focusing on the various formulations of the drug, its dosage, drug delivery, and duration of therapy. The study also covers the common outcomes of steroid therapy and its side effects.

## Introduction and background

Eosinophilic esophagitis (EoE) is a chronic esophagus disorder characterized by the eosinophil infiltration of the esophagus [1]. It is an antigen-mediated condition [1]. At present, the disease burden is estimated to be 50-100 per 100,000 people globally [2]. The incidence is 5-10 cases per 100,000 people annually [2]. Recent studies have shown an increase in both incidence and prevalence due to the rising awareness of the disease [3].

EoE has a varying clinical presentation in children and adults, ranging from nonspecific symptoms such as vomiting, feed refusal, and regurgitation in children to pain and dysphagia in adults [4-6]. Furthermore, if untreated, it can lead to esophageal stricture and food impaction [7]. Diagnosis requires the presence of 15 eosinophils per high power field (hpf) on esophageal biopsy [8].

Owing to the chronic nature of the disease, early identification and treatment are of utmost importance to prevent the consequences of untreated EoE. There exists a discordance between clinical features and disease severity (the disease is histologically and endoscopically active in untreated cases, even in the absence of symptoms) [1,9]. It has also been shown that the duration of untreated or undiagnosed disease correlates with stricture formation, ranging from 17% of strictures forming in two years to 71% in 20 years [9]. EoE has a significant impact on the quality of life of patients and family members due to the restriction of social activities, leading to emotional distress [10].

EoE can be treated using both noninvasive and invasive methods. It is treated by dietary elimination of certain food items with gradual reintroduction, proton pump inhibitor (PPI) therapy, local or systemic corticosteroid therapy, and stricture management through esophageal dilation [1,11].

The steroid preparations used in the treatment vary from inhaled formulations primarily used in the treatment of asthma to oral viscous preparations with local action in the esophagus. This study aims to identify the various steroid preparations available for treatment and compare these preparations based on the drug used, dosage, duration of therapy, and mode of delivery.

## Review

Pathophysiology and the role of steroids

EoE is a chronic immune-related condition predominantly observed in males, with a male-to-female ratio of 3:1 [12]. Pathophysiology involves a complex interplay of genetic, epigenetic, immunological, and environmental factors [1,13,14]. Various studies have shown the role of T-helper 2 (Th2) cells in the recruitment of eosinophils, basophils, and mast cells to the esophagus via mediators such as interleukin (IL)-4, IL-5, and IL-13 [15]. Once recruited, the eosinophils release proteins and mediators of inflammation such as major basic protein (MBP), eosinophilic cationic protein (ECP), tumor growth factor (TGF) beta, platelet-activating factor, and IL-13 [16]. These proteins and mediators cause mucosal damage, remodeling, and fibrosis, leading to stricture formation.

Steroids are used as the first-line therapy, along with PPI and dietary modification. Steroids can cause remission of inflammation in the esophagus by inhibiting the release of proinflammatory cytokines [8,17]. In 1998, Liacouras et al. established the effectiveness of systemic steroid treatment [18]. Later, various studies were conducted to determine the efficacy of topical steroids delivered via a metered-dose inhaler [19].

**Figure 1:**
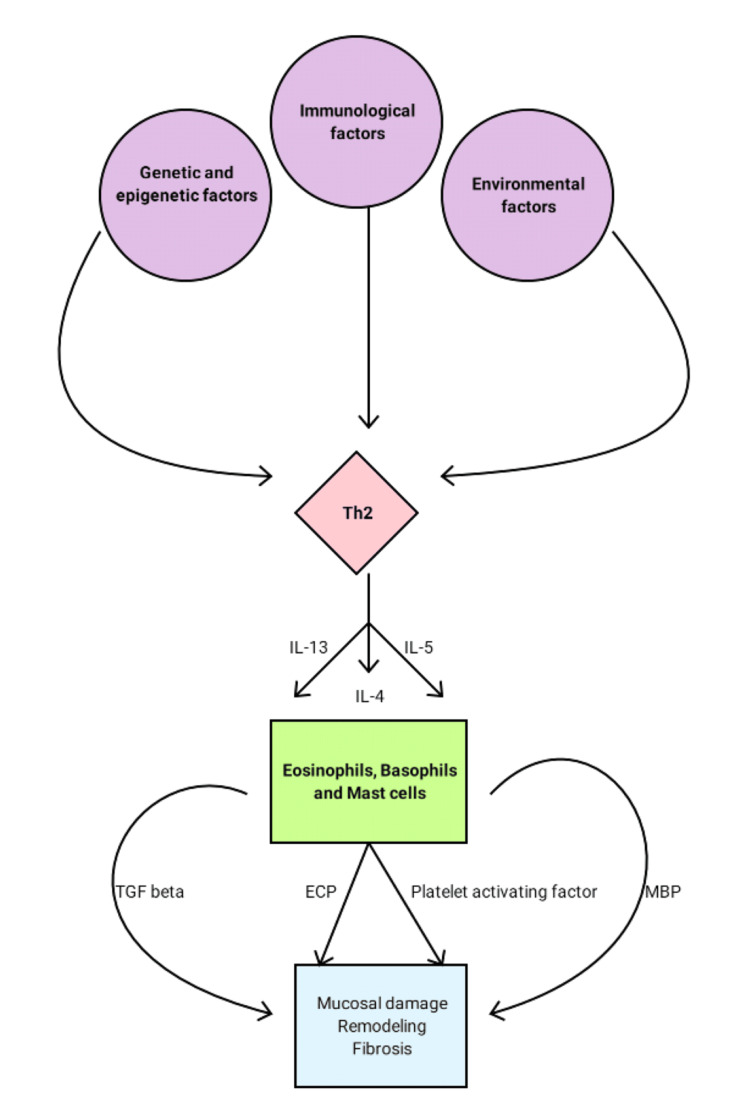
Pathophysiology of EoE Th2: T-helper 2 cell; IL: interleukin; TGF: tumor growth factor; ECP: eosinophilic cationic protein; MBP: major basic protein Prepared by Ramanan, Sruthi. Data taken from [13-16]

Recently, fluticasone, budesonide, and ciclesonide have been the coveted steroids used in the treatment; however, there is no consensus on the choice of drug, dose, mode of delivery, or duration of treatment for the effective resolution and maintenance of EoE. The pathophysiology is presented in Figure 1.

Literature overview from the last decade

In the last decade, several studies have emerged that compared the effectiveness of various treatment options in terms of histological remission and symptomatic improvement in patients with EoE. For this review article, only studies that compared the efficacy of steroids against placebo or other steroids are included.

In 2012, Schroeder et al. treated four children with inhaled ciclesonide for eight weeks [20]. Ciclesonide was chosen as the drug of choice in their study as it was previously shown to be effective in treating allergic conditions at a dose similar to or lesser than that of fluticasone and budesonide [21,22]. Moreover, ciclesonide was hypothesized to have higher activity at the mucosal surface, thereby decreasing the systemic side effects of the drug [22,23]. The study showed that ciclesonide successfully induced remission in all four children both histologically and clinically. Moreover, histological features such as edema, basal cell hyperplasia, and spongiosis had decreased following treatment with ciclesonide [20].

The first study comparing the effectiveness of two different delivery methods of budesonide was conducted by Dellon et al. in 2012. In their study, Dellon et al. compared the effectiveness of 1 mg of budesonide that was nebulized and then swallowed to an oral viscous solution of budesonide. Using scintigraphy, it was found that the esophageal contact time was higher for the oral viscous formulation [24]. This higher contact time also correlated to a more significant improvement in histological features. However, the dysphagia score improved equally in patients, irrespective of the mode of delivery. Esophageal candidiasis developed in 14% of patients who took oral viscous budesonide but were asymptomatic [24].

The efficacy of fluticasone in treatment was highlighted by the study conducted by Faubion et al. involving four pediatric patients [19]. However, Konikoff et al., in their study, showed that only 50% of patients treated with fluticasone (880 mg) achieved remission successfully [25]. In 2014, Butz et al. conducted a study to demonstrate the effectiveness of high-dose fluticasone (1760 mg) and the effect of dose reduction following high-dose fluticasone [26]. Of the 42 study subjects, 28 received high-dose fluticasone, and 14 received a placebo at the end of three months. They found that 65% of patients in the treatment group were in complete remission compared to 0% in the placebo group. Following this, the patients who reached complete remission received a 50% reduced dose of fluticasone for three months, and those who were not in complete remission received high-dose fluticasone for another three months. At the end of six months, 65%-77% of patients who had received high doses of fluticasone for at least three months reached histological remission, and 50% dose reduction was effective in approximately 73%-93% of patients [26].

In 2015, Miehlke et al. compared the efficacy of two different formulations of budesonide: effervescent tablets and an oral viscous solution. In this study, 76 subjects were divided into four groups and treated with a 14-day course of budesonide effervescent tablet 1 mg BD (BET1), budesonide effervescent tablet 2 mg BD (BET2), budesonide oral viscous solution, or placebo [27]. The study showed that treatment with budesonide was effective when compared with placebo. Additionally, the study subjects preferred budesonide effervescent tablets over oral solutions [27].

In 2016, Andreae et al. conducted a study to evaluate the long-term effects of fluticasone on pediatric patients with EoE. The mean follow-up period was 20.4 months. The patients were instructed to swallow two puffs of fluticasone twice daily of varying strengths depending on the age of the patient [28]. The primary outcome assessed was eosinophil count per high power field. Secondary outcomes such as histological and clinical scores were also calculated. This study showed that fluticasone was effective for the long-term management of pediatric patients diagnosed with EoE with minimal safety concerns [28]. The details of the studies in this review are summarized in Table 1.

**Table 1:** The four Ds of steroid therapy: drug, dose, delivery, and duration Eos/hpf: eosinophil count per high power field; URI: upper respiratory infections

Study/year	Subjects	Drug	Dose	Delivery	Duration	Outcome studied	Side effects
Schroeder et al., 2012 [20]	4	Ciclesonide	80-160 mcg	Inhaled	8 weeks	Eos/hpf	-
Dellon et al., 2012 [24]	22	Budesonide	1 mg	Nebulized/oral viscous	8 weeks	Eosinophil count, dysphagia score	Candidiasis
Butz et al., 2014 [26]	42	Fluticasone	1760 or 880 mcg	Oral	3 months	Eos/hpf	Oral candidiasis, low serum cortisol
Miehlke et al., 2015 [27]	76	Budesonide	1 mg/2 mg/5 mg BD	Effervescent tablet (1 and 2 mg)/viscous solution (5 mg)	14 days	Endoscopic score, histology, dysphagia	Candidiasis, lip edema
Andreae et al., 2016 [28]	54	Fluticasone	2 puffs of varying strength BD	Nebulized then swallowed	20-68 months	Clinical score, histological score, endoscopic score	Esophageal candidiasis
Dellon et al., 2017 [29]	93	Budesonide	2 mg BD	Oral	12 weeks	Dysphagia score, Eos/hpf	Candidiasis, URI, withdrawal symptoms
Dellon et al., 2019 [30]	129	Budesonide Fluticasone	1 mg/4 ml BD, 880 mcg BD	Viscous solution, inhaled	8 weeks, 8 weeks	Eos/hpf, dysphagia score	Esophageal candidiasis, oral candidiasis
Lucendo et al., 2019 [31]	88	Budesonide	1 mg BD	Oral	6 weeks	Eos/hpf, dysphagia score, odynophagia score	Candidiasis
Dellon et al., 2019 [32]	82	Budesonide	2 mg+/- 2 mg	Oral	24 weeks	Eos/hpf, mean eosinophil count	Candidiasis, low serum cortisol
Olivia et al., 2019 [33]	20	Budesonide	2-4 mg, 1-2 mg	Oral, oral	12 weeks, 12 weeks	Eos/hpf	
Straumann et al., 2020 [34]	204	Budesonide	0.5 mg BD, 1 mg BD	Oral, oral	48 weeks, 48 weeks	Maintenance of remission	Candidiasis, low serum cortisol
Song et al., 2020 [35]	22	Budesonide	2 mg, 9 mg	Oral viscous, gelatin capsule	Single dose		

In 2017, Dellon et al. conducted a study to assess the efficacy of oral viscous budesonide preparation by measuring the patient-reported symptom improvement using the dysphagia score and histological response as the primary outcomes [29]. The study concluded that oral viscous budesonide preparation is an effective treatment in adolescents and adults compared to placebo [29].

In 2019, four different studies evaluating the safety and efficacy of topical steroids emerged [30-33]. These studies, however, used varying treatment regimens. In the study conducted by Dellon et al., they compared the efficacy of oral viscous budesonide to inhaled fluticasone in search of a viable first-line treatment option. However, the study reported that the histological and symptomatic improvements were comparable in all groups; hence, both treatment routes were effective.

Lucendo et al. compared the efficacy of budesonide oral tablets to placebo. They reported that 58% of the subjects taking the tablets achieved complete remission at six weeks, and 85% achieved complete remission by 12 weeks [31].

The studies described in this review have primarily focused on the ability of steroids to induce remission in patients with EoE. However, two of these studies advocated for the use of fluticasone as a maintenance therapy for patients [27,28]. Dellon et al. performed a study as a continuation of a previous placebo-controlled trial testing the efficacy of oral budesonide in adolescents and adults [29]. The subjects who participated in that study were enrolled in a 24-week open-labeled trial to measure the safety and efficacy of oral budesonide suspension as a maintenance therapy. In this study, the patients were administered 2 mg of budesonide for the first 12 weeks, followed by 2 mg/2 mg + 1.5-2 mg for the next 12 weeks. Budesonide oral suspension was a well-tolerated drug with sustained remission over 24 weeks [32].

While the study conducted by Dellon et al. measured the safety and efficacy of oral budesonide as a maintenance therapy in adolescents and adults [32], Olivia et al. conducted a study to assess the efficacy in the pediatric population. In this study, 20 patients were enrolled and given an initial dose of 2-4 mg of budesonide. A total of 18 children achieved remission at the end of 12 weeks. Subsequently, they were given half the initial dose and evaluated at 24 and 36 weeks. At 24 weeks, 85% of the patients were in remission and 45% at 36 weeks [33].

The studies conducted by Dellon et al. and Olivia et al. were followed by Straumann et al. in 2020, who conducted a double-blinded randomized control trial comparing the efficacy of budesonide 0.5 mg, 1 mg, and placebo in maintaining remission for 48 weeks. Their study showed that budesonide was effective in maintaining remission compared to placebo, with 73.5% maintaining remission with 0.5 mg budesonide and 75% with 1 mg [34].

Despite multiple drugs being used to treat EoE, no drug is approved by the US FDA. Song et al. conducted a study comparing the systemic effects of budesonide oral solution to Entocort EC, which is an FDA-approved formulation for Crohn's disease [35]. The study showed that the systemic effects from 2 mg oral budesonide solution are lesser than those of Entocort EC 9 mg. Thus, 2 mg of oral budesonide is a safer option for treating EoE.

Patient demographics and study characteristics

Although EoE is prevalent in all age groups, it is commonly a disorder in children and adolescents. In adults, it usually presents before the age of 50 years [1,36]. The age range of the participants in the studies discussed in this review varied from 7.75 to 39.7 years. EoE affects males three times more than females [1,37]. This is consistent with the demographics of most of the studies in this review, except the four studies conducted by Dellon et al. [24,29,30,32].

The studies included in this review ranged from case series [20] to multicenter placebo-controlled RCTs [26]. The case series conducted by Schroeder et al. had the least number of subjects, while the RCT conducted by Straumann et al. had the largest sample size, consisting of 204 subjects [20,34]. This review also included the study by Song et al., which is the only study to include healthy volunteers rather than patients [35]. The demographic details are demonstrated in Table 2.

**Table 2:** Demographic data and study characteristics M: male; F: female

Study/year	Subjects	Mean age	Gender	Study type
Schroeder et al., 2012 [20]	4	6.75	M: 3, F: 1	Case series
Dellon et al., 2012 [24]	22	35	M: 13, F: 9	Randomized control trial
Butz et al., 2014 [26]	42	12.85	M: 35, F: 7	Randomized, multisite, double-blind, placebo-controlled trial
Miehlke et al., 2015 [27]	76	39.7+/-13.1	M: 63, F: 13	Double-blind, double-dummy, randomized, placebo-controlled, multicenter trial
Andreae et al., 2016 [28]	54	6.5	M: 43, F: 11	Open-label, prospective, single-center study
Dellon et al., 2017 [29]	93	-	M: 64, F: 29	Multicenter, randomized, double-blind, placebo-controlled, parallel-group phase 2 trial
Dellon et al., 2019 [30]	129	-	M: 74, F: 55	Randomized, double-blind, double-dummy, parallel-arm, single-center, superiority clinical trial
Lucendo et al., 2019 [31]	88	37	M: 73, F: 15	Randomized double-blinded, placebo-controlled, parallel study
Dellon et al., 2019 [32]	82	-	M: 58, F: 24	Multicenter, randomized, double-blind, placebo-controlled trial
Oliva et al., 2019 [33]	20	10	M: 15, F: 5	Prospective, single-site, pilot study
Straumann et al., 2020 [34]	204	36+/-10.6	M: 169, F: 35	Multicentered, randomized, double-blinded, placebo-controlled trial, phase III trial
Song et al., 2020 [35]	22	39.2+/-10.2	M: 11, F: 11	Open-labeled, single-center, crossover study

Drug data

The studies in this review used three topical steroid preparations, namely, ciclesonide, budesonide, and fluticasone. While ciclesonide was used in only one study [20], budesonide was used in nine studies [24,27,29,30-35]. Among all the studies, ciclesonide and fluticasone were either inhaled or nebulized and then swallowed [26,28], whereas there were three different preparations for budesonide. Budesonide was used orally (as a viscous solution, tablet, and nebulized then swallowed) and also available as a gelatin capsule [24,27]. However, the dosages of the drugs varied, particularly in the pediatric population. With the given data, the lowest dose of budesonide used was 0.5 mg BD, and the highest was 4 mg BD orally [32,34] (although the gelatin capsule was 9 mg OD). The dose of fluticasone was either low dose (880 mcg) or high dose (1760 mcg) [26].

Outcomes studied and side effect profile

The primary outcome in the majority of studies is disease remission assessed using clinical and histological parameters such as the dysphagia scale and the number of eosinophils per high power field, respectively [20,24,26-28]. Alternatively, the primary endpoint was the maintenance of remission again assessed using histological and clinical parameters [34]. An exception was the study conducted by Song et al. regarding the safety and pharmacological properties of the drug.

The most common side effect reported was candida infections of the oral cavity and esophagus. All the studies reported that the infection was asymptomatic and resolved with the use of antifungal medication. Three studies additionally reported low serum cortisol concentration as a side effect, which was asymptomatic [26,32,34].

Limitations

This review article focuses solely on steroids as a treatment option for EoE, excluding other options such as dietary changes and proton pump inhibitors, thereby not accounting for the effect of combination therapy. Moreover, the study has not accounted for steroid resistance, which is a common cause of nonresponse to steroid therapy.

## Conclusions

EoE is a chronic immune-mediated disorder of the esophagus due to the infiltration of eosinophils. The primary modalities of treatment are an elimination diet, drugs such as PPI and topical steroids, and esophageal dilation in cases of stricture formation. Currently, there is no FDA-approved drug for the treatment. In this review article, various steroid preparations were compared to one another for treatment. However, there is no consensus on the drug, its dose, route of administration, or treatment duration. This situation calls for a large-scale trial comparing various formulations of different steroids to come to a consensus regarding the drug, its dose, and the route of administration that is best suited for the treatment.

## References

[1] Liacouras CA, Furuta GT, Hirano I (2011). Eosinophilic esophagitis: updated consensus recommendations for children and adults. J Allergy Clin Immunol.

[2] Dellon ES, Hirano I (2018). Epidemiology and natural history of eosinophilic esophagitis. Gastroenterology.

[3] Syed AA, Andrews CN, Shaffer E, Urbanski SJ, Beck P, Storr M (2012). The rising incidence of eosinophilic oesophagitis is associated with increasing biopsy rates: a population-based study. Aliment Pharmacol Ther.

[4] Liacouras CA, Spergel JM, Ruchelli E (2005). Eosinophilic esophagitis: a 10-year experience in 381 children. Clin Gastroenterol Hepatol.

[5] Noel RJ, Putnam PE, Rothenberg ME (2004). Eosinophilic esophagitis. N Engl J Med.

[6] Straumann A, Katzka DA (2018). Diagnosis and treatment of eosinophilic esophagitis. Gastroenterology.

[7] Warners MJ, Oude Nijhuis RA, de Wijkerslooth LR, Smout AJ, Bredenoord AJ (2018). The natural course of eosinophilic esophagitis and long-term consequences of undiagnosed disease in a large cohort. Am J Gastroenterol.

[8] Dellon ES, Gonsalves N, Hirano I, Furuta GT, Liacouras CA, Katzka DA (2013). ACG clinical guideline: evidenced based approach to the diagnosis and management of esophageal eosinophilia and eosinophilic esophagitis (EoE). Am J Gastroenterol.

[9] Schoepfer AM, Safroneeva E, Bussmann C, Kuchen T, Portmann S, Simon HU, Straumann A (2013). Delay in diagnosis of eosinophilic esophagitis increases risk for stricture formation in a time-dependent manner. Gastroenterology.

[10] Lucendo AJ, Arias-González L, Molina-Infante J, Arias Á (2017). Systematic review: health-related quality of life in children and adults with eosinophilic oesophagitis-instruments for measurement and determinant factors. Aliment Pharmacol Ther.

[11] Lucendo AJ, Molina-Infante J, Arias Á (2017). Guidelines on eosinophilic esophagitis: evidence-based statements and recommendations for diagnosis and management in children and adults. United European Gastroenterol J.

[12] Mansoor E, Cooper GS (2016). The 2010-2015 prevalence of eosinophilic esophagitis in the USA: a population-based study. Dig Dis Sci.

[13] Alexander ES, Martin LJ, Collins MH (2014). Twin and family studies reveal strong environmental and weaker genetic cues explaining heritability of eosinophilic esophagitis. J Allergy Clin Immunol.

[14] Lim EJ, Lu TX, Blanchard C, Rothenberg ME (2011). Epigenetic regulation of the IL-13-induced human eotaxin-3 gene by CREB-binding protein-mediated histone 3 acetylation. J Biol Chem.

[15] Rosenberg HF, Dyer KD, Foster PS (2013). Eosinophils: changing perspectives in health and disease. Nat Rev Immunol.

[16] Travers J, Rothenberg ME (2015). Eosinophils in mucosal immune responses. Mucosal Immunol.

[17] Greenhawt M, Aceves SS, Spergel JM, Rothenberg ME (2013). The management of eosinophilic esophagitis. J Allergy Clin Immunol Pract.

[18] Liacouras CA, Wenner WJ, Brown K, Ruchelli E (1998). Primary eosinophilic esophagitis in children: successful treatment with oral corticosteroids. J Pediatr Gastroenterol Nutr.

[19] Faubion WA Jr, Perrault J, Burgart LJ, Zein NN, Clawson M, Freese DK (1998). Treatment of eosinophilic esophagitis with inhaled corticosteroids. J Pediatr Gastroenterol Nutr.

[20] Schroeder S, Fleischer DM, Masterson JC, Gelfand E, Furuta GT, Atkins D (2012). Successful treatment of eosinophilic esophagitis with ciclesonide. J Allergy Clin Immunol.

[21] Stoeck M, Riedel R, Hochhaus G (2004). In vitro and in vivo anti-inflammatory activity of the new glucocorticoid ciclesonide. J Pharmacol Exp Ther.

[22] Pearlman DS, Berger WE, Kerwin E, Laforce C, Kundu S, Banerji D (2005). Once-daily ciclesonide improves lung function and is well tolerated by patients with mild-to-moderate persistent asthma. J Allergy Clin Immunol.

[23] Mutch E, Nave R, McCracken N, Zech K, Williams FM (2007). The role of esterases in the metabolism of ciclesonide to desisobutyryl-ciclesonide in human tissue. Biochem Pharmacol.

[24] Dellon ES, Sheikh A, Speck O (2012). Viscous topical is more effective than nebulized steroid therapy for patients with eosinophilic esophagitis. Gastroenterology.

[25] Konikoff MR, Noel RJ, Blanchard C (2006). A randomized, double-blind, placebo-controlled trial of fluticasone propionate for pediatric eosinophilic esophagitis. Gastroenterology.

[26] Butz BK, Wen T, Gleich GJ (2014). Efficacy, dose reduction, and resistance to high-dose fluticasone in patients with eosinophilic esophagitis. Gastroenterology.

[27] Miehlke S, Hruz P, Vieth M (2016). A randomised, double-blind trial comparing budesonide formulations and dosages for short-term treatment of eosinophilic oesophagitis. Gut.

[28] Andreae DA, Hanna MG, Magid MS, Malerba S, Andreae MH, Bagiella E, Chehade M (2016). Swallowed fluticasone propionate is an effective long-term maintenance therapy for children with eosinophilic esophagitis. Am J Gastroenterol.

[29] Dellon ES, Katzka DA, Collins MH, Hamdani M, Gupta SK, Hirano I (2017). Budesonide oral suspension improves symptomatic, endoscopic, and histologic parameters compared with placebo in patients with eosinophilic esophagitis. Gastroenterology.

[30] Dellon ES, Woosley JT, Arrington A (2019). Efficacy of budesonide vs fluticasone for initial treatment of eosinophilic esophagitis in a randomized controlled trial. Gastroenterology.

[31] Lucendo AJ, Miehlke S, Schlag C (2019). Efficacy of budesonide orodispersible tablets as induction therapy for eosinophilic esophagitis in a randomized placebo-controlled trial. Gastroenterology.

[32] Dellon ES, Katzka DA, Collins MH, Gupta SK, Lan L, Williams J, Hirano I (2019). Safety and efficacy of budesonide oral suspension maintenance therapy in patients with eosinophilic esophagitis. Clin Gastroenterol Hepatol.

[33] Oliva S, Rossetti D, Papoff P (2019). A 12-week maintenance therapy with a new prepared viscous budesonide in pediatric eosinophilic esophagitis. Dig Dis Sci.

[34] Straumann A, Lucendo AJ, Miehlke S (2020). Budesonide orodispersible tablets maintain remission in a randomized, placebo-controlled trial of patients with eosinophilic esophagitis. Gastroenterology.

[35] Song IH, Finkelman RD, Lan L (2020). A pharmacokinetic bridging study to compare systemic exposure to budesonide between budesonide oral suspension and ENTOCORT EC in healthy individuals. Drugs R D.

[36] Spergel JM, Brown-Whitehorn TF, Beausoleil JL, Franciosi J, Shuker M, Verma R, Liacouras CA (2009). 14 years of eosinophilic esophagitis: clinical features and prognosis. J Pediatr Gastroenterol Nutr.

[37] Prasad GA, Alexander JA, Schleck CD (2009). Epidemiology of eosinophilic esophagitis over three decades in Olmsted County, Minnesota. Clin Gastroenterol Hepatol.

